# PRED_PPI: a server for predicting protein-protein interactions based on sequence data with probability assignment

**DOI:** 10.1186/1756-0500-3-145

**Published:** 2010-05-26

**Authors:** Yanzhi Guo, Menglong Li, Xuemei Pu, Gongbin Li, Xuanmin Guang, Wenjia Xiong, Juan Li

**Affiliations:** 1 College of Chemistry, Sichuan University, Chengdu 610064, PR China

## Abstract

**Background:**

Protein-protein interactions (PPIs) are crucial for almost all cellular processes, including metabolic cycles, DNA transcription and replication, and signaling cascades. Given the importance of PPIs, several methods have been developed to detect them. Since the experimental methods are time-consuming and expensive, developing computational methods for effectively identifying PPIs is of great practical significance.

**Findings:**

Most previous methods were developed for predicting PPIs in only one species, and do not account for probability estimations. In this work, a relatively comprehensive prediction system was developed, based on a support vector machine (SVM), for predicting PPIs in five organisms, specifically humans, yeast, *Drosophila*, *Escherichia coli*, and *Caenorhabditis elegans*. This PPI predictor includes the probability of its prediction in the output, so it can be used to assess the confidence of each SVM prediction by the probability assignment. Using a probability of 0.5 as the threshold for assigning class labels, the method had an average accuracy for detecting protein interactions of 90.67% for humans, 88.99% for yeast, 90.09% for *Drosophila*, 92.73% for *E. coli*, and 97.51% for *C. elegans*. Moreover, among the correctly predicted pairs, more than 80% were predicted with a high probability of ≥0.8, indicating that this tool could predict novel PPIs with high confidence.

**Conclusions:**

Based on this work, a web-based system, Pred_PPI, was constructed for predicting PPIs from the five organisms. Users can predict novel PPIs and obtain a probability value about the prediction using this tool. Pred_PPI is freely available at http://cic.scu.edu.cn/bioinformatics/predict_ppi/default.html.

## Background

Protein-protein interactions (PPIs) are essential for almost all cellular processes. Currently, PPIs discovered by experimental methods are absolutely insufficient for examining the complete PPI networks [1]. Consequently, computational tools for effectively identifying PPIs are increasingly important. Current computational methods can be classified into two main approaches. The first is based on genomic [2] or structural information of proteins [3,4]. However, these methods cannot be implemented if prior information about the proteins is not available. The second approach is based on protein primary sequences [5-7].

In general, a PPI predictor should be able to provide the probability estimation for its prediction in the output. However, most methods for PPI prediction were developed for only one particular species, and do not include a probability estimation. The sequence-based method proposed by Guo et al. [7] yields a good performance when applied to predicting PPIs of *Saccharomyces cerevisiae*. Therefore, we extended the application of the method to additional organisms. PPI prediction models were constructed for humans, yeast, *Drosophila*, *Escherichia coli*, and *Caenorhabditis elegans*, with a probability assignment for each support vector machine (SVM) prediction. The web-server Pred_PPI was developed for free use to predict novel PPIs with probability assignments.

## Materials and methods

Interaction information for human proteins was from the Human Protein References Database (HPRD), release 7_20070901 [8]. The PPI data for yeast, *Drosophila*, *E. coli*, and *C. elegans *were from the Database of Interacting Proteins (DIP), version DIP_20070219 [9]. After removing protein pairs that contained a protein of less than 50 amino acids, 37027 PPIs remained in the dataset for humans, 5943 for yeast, 22975 for *Drosophila*, 6954 for *E. coli*, and 4030 for *C. elegans*. Noninteracting pairs were determined based on protein subcellular localization information, as described by Guo et al. [7]. Negative datasets were built, and the number of negative pairs was equal to the positive pairs. For each organism, the entire dataset was partitioned into a training set and a test set (detailed description in Additional File 1). To minimize the data dependence on the prediction model, the sampling process was repeated five times, generating five training sets and five test sets. Each model was evaluated by averaging the prediction results of the five test sets.

Classifications were implemented using libsvm 2.84 [10]. This software predicts class label and probability information. Details about the method of extending SVM for probability estimates are in Wu et al. [11]. Choosing radial basic function as the kernel function, two parameters, the regularization parameter *C*, and the kernel width parameter γ were optimized using a grid search approach.

## Results and Discussion

For two-class problem, the prediction results were obtained from libsvm, using a default probability threshold of 0.5. Using this threshold, the prediction results for the test sets for of each species are in Table 1. This method produced PPI prediction models with accuracies of 90.67 ± 0.17% for humans, 88.99 ± 0.75% for yeast, 90.09 ± 8.39% for *Drosophila*, 92.73 ± 3.94% for *E. coli*, and 97.51 ± 0.22% for *C. elegans*, indicating a powerful prediction ability, and general applicability. The optimal values of *C *and γ are in Table S1 (Additional File 2). Using these optimal values, each predictor was constructed based on the entire dataset.

**Table 1:** Prediction results of the test sets for five organisms with probability threshold of 0.5.

A. For *Human *PPI prediction			
**Test set**	**Sensitivity (%)**	**Specificity (%)**	**Accuracy (%)**

1	88.91	92.13	90.67
2	89.05	92.48	90.76
3	89.34	92.03	90.69
4	89.24	92.42	90.83
5	89.28	91.49	90.39
Average	89.17	92.17	90.67 ± 0.17

**B. For *Yeast *PPI prediction**			

**Test set**	**Sensitivity (%)**	**Specificity (%)**	**Accuracy (%)**
1	87.89	89.19	88.54
2	88.14	89.78	88.96
3	89.36	89.15	89.26
4	86.84	89.40	88.12
5	88.65	91.55	90.10
Average	88.17	89.81	88.99 ± 0.75

**C. For *Drosophila *PPI prediction**			

**Test set**	**Sensitivity (%)**	**Specificity (%)**	**Accuracy (%)**
1	99.15	91.19	95.17
2	99.33	63.66	81.50
3	99.80	92.75	96.28
4	99.63	94.63	97.13
5	99.76	61.03	80.39
Average	99.53	80.65	90.09 ± 8.39

**D. For *E.coli *PPI prediction**			

**Test set**	**Sensitivity (%)**	**Specificity (%)**	**Accuracy (%)**
1	91.27	97.87	94.55
2	96.55	91.55	94.05
3	93.42	96.87	95.15
4	98.49	71.28	84.88
5	95.83	94.18	95.00
Average	95.11	90.35	92.73 ± 3.94

**E. For *C.elegans *PPI prediction**			

**Test set**	**Sensitivity (%)**	**Specificity (%)**	**Accuracy (%)**
1	97.02	98.20	97.91
2	96.53	98.76	97.33
3	96.34	99.13	97.74
4	96.34	98.33	97.33
5	96.09	98.33	97.21
Average	96.46	98.55	97.51 ± 0.22

With probability 0.5 as the threshold to assign class label, the prediction results of each species are shown in this table. For all species, the sensitivities are >88%, the specificities are >80% and the prediction accuracies are >88%. Moreover, each model gives a relatively low standard deviation (SD) of no more than 10%. So this method has a good robustness.

**Figure 1:**
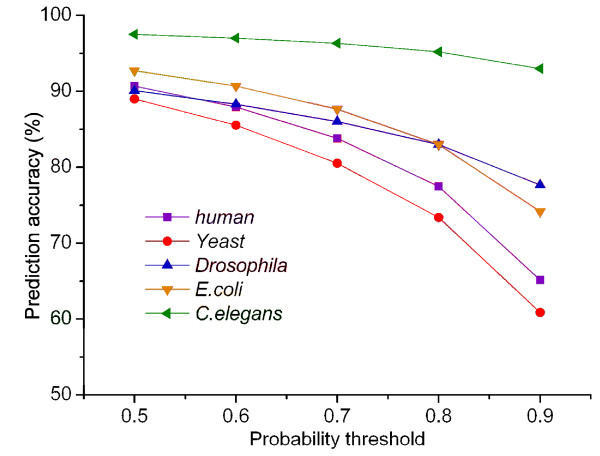
**Curves of prediction accuracy versus probability threshold**. The figure shows the average prediction accuracy of the method under the different probability thresholds of 0.5, 0.6, 0.7, 0.8 and 0.9 respectively. For predictors of five species, the total prediction accuracy was obtained by averaging those of five test sets.

**Figure 2:**
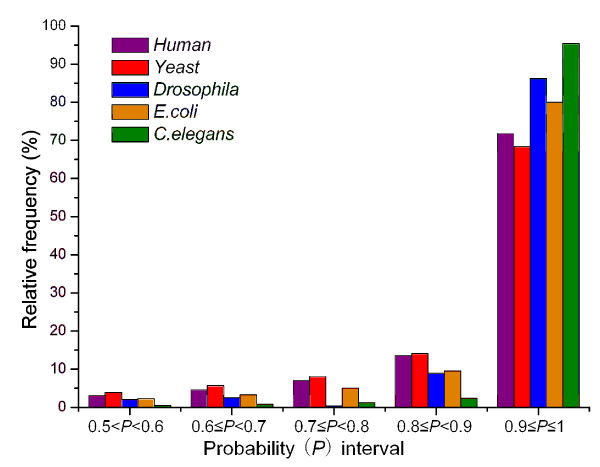
**The frequency distributions of the correctly predicted samples within different probability intervals**. Among the correctly predicted samples under the default probability threshold of 0.5, the relative frequency distributions of them within different probability intervals are represented by this figure.

The PPIs from STRING http://string.embl.de[12] were classified into three categories: high, medium, and low confidence, using STRING scores. Confidence can be defined as the probability of an interaction between two proteins. Thus, users can select PPIs with a particular confidence level. To assess the confidence level of PPIs predicted by our method, probability thresholds of 0.6, 0.7, 0.8, and 0.9 were selected for assigning class labels. The curves of prediction accuracy versus probability threshold are in Figure 1. Under rigorous restriction of prediction probability ≥0.8, the lowest prediction accuracy was still >70% when the probability threshold was 0.8, and >60% when the probability threshold was 0.9. The detailed results for the five species, using probability thresholds of 0.5, 0.6, 0.7, 0.8 and 0.9 are in Table S2 (Additional File 2). In addition, the prediction probability values of the correctly predicted samples could be divided into five different intervals: (0.5, 0.6), [0.6, 0.7), [0.7, 0.8), [0.8, 0.9) and [0.9, 1]. For additional data on the confidence level of the predictions, the frequency distributions of correctly predicted samples within different probability intervals were determined (Figure 2). Among the correctly predicted pairs, the overwhelming majority of predictions (>80%) were within the probability interval of [0.8, 1], and more than 65% were predicted with a probability ranging from 0.9 to 1. This indicated that the PPIs were predicted by our method with high confidence. Additional data are in Table S3 (Additional File 2).

Finally, to further verify the general performance of this method, a test set of human PPIs was constructed. Recently published data was collected from HPRD Release 8_20090706, by excluding PPIs from HPRD release 7_20070901. The test set contained 2201 PPIs that were not included in the entire training set. For predicting human PPIs, the Shen et al. method [6] achieved the highest accuracy, with 83.9%. Therefore, we used this test set for an unbiased evaluation of the method developed here, and the Shen et al. method [6]. Comparison results are in Table S4 (Additional File 2). Using the default probability threshold of 0.5, 2106 PPIs were correctly predicted by our method with a prediction accuracy of 93.59%. The Shen et al. method [6] predicted 1479, with an accuracy of only 66.88%. Moreover, among the correctly predicted PPIs, 89.50% (1885 PPIs) had a high interaction probability of ≥0.9 by our method, while only 66.78% were predicted with ≥0.9 interaction probability by the Shen et al. method [6]. To avoid homology bias in the prediction result, all proteins in the test set were aligned with those in the training set using the BLASTCLUST program [13]. We removed protein pairs in the test set with a ≥25% pairwise sequence identity to those in the training set. The remaining 1983 PPIs comprised an independent dataset. The prediction results of our method using this independent dataset are also in Table S4 (Additional File 2). The method still achieved a high accuracy of 93.09% for the independent dataset, and 90% of the correctly predicted PPIs had a ≥0.9 interaction probability. These results indicated that the newly developed method not only provided a powerful general performance, but also gave high-confidence predictions.

## Web server for PPI prediction

**Figure 3:**
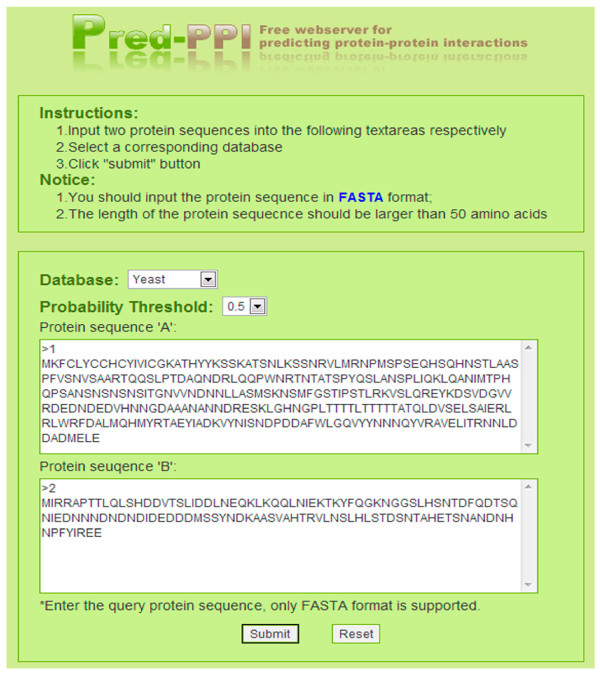
**Screen shot of the input page of Pred_PPI**. This figure shows how the users use the web server to input the query proteins 'A' and 'B' whose interaction needs to be predicted. Before submitting, users should select the respective predictor of one species that the query proteins belong to.

**Figure 4:**
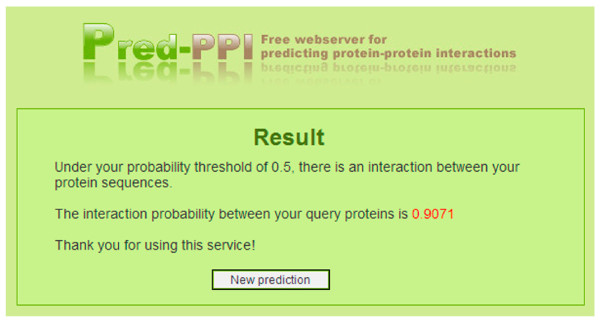
**Screen shot of the output page of Pred_PPI**. This figure shows the prediction result in the output page. The user will get the actual interaction probability between the query proteins.

The interaction prediction server, Pred_PPI is freely available to any researcher wishing to use it for non-commercial purposes. First, users select an organism-specific predictor for the species corresponding to the query proteins. Then, a probability threshold is chosen for the classifications, with a default probability threshold of 0.5. The inputs to the prediction server are protein sequences "A" and "B", whose interaction is to be predicted. A screenshot of the input page is shown in Figure 3 and a screen shot of the result page is in Figure 4. The prediction result reports whether the query proteins interact under the selected probability threshold, and provides the actual probability value of the prediction.

## Competing interests

The authors declare that they have no competing interests.

## Authors' contributions

YG was involved in the design of the study; carried out the construction of predictors, analyzed results and wrote the manuscript. ML and XP helped doing the statistical analysis and approved the final version. GL designed and constructed the web-server. XG, WX and JL helped collecting PPI data and doing the statistical analysis. All authors have read and approved the final manuscript.

## Supplementary Material

Additional file 1 **Data set partition**. Additional File 1 gives the detailed description about the partitioning process of the training set and test set for the whole dataset of each organism. Click here for file

Additional file 2 **Detailed prediction results**. Additional File 2 is the supplemental data file providing detailed prediction results of the predictors built in this paper. It includes four tables. Table S1 lists the optimal values of C and γ for the predictors of five organism species. Table S2 shows the average prediction results for five organisms with probability threshold of 0.5, 0.6, 0.7, 0.8 and 0.9 respectively. Table S3 demonstrates the frequency distributions of the correctly predicted samples within different probability intervals and Table S4 shows the prediction performance of our method on the test set. Click here for file
