# Supplementary material for: PRED_PPI: a server for predicting protein-protein interactions based on sequence data with probability assignment
Source: BMC Res Notes. 2010 May 26;3:145. doi: 10.1186/1756-0500-3-145 (PMC2883990; doi:10.1186/1756-0500-3-145)
Supplement: Additional file 1 — Data set partition. Additional File 1 gives the detailed description about the partitioning process of the training set and test set for the whole dataset of each organism. [file 1756-0500-3-145-S1.DOC]

**Dataset partition**

The number of PPIs in the final datasets for humans, yeast, *Drosophila*, *E.coli*, *C.elegans* is 74054, 11886, 43950, 13908, 8060 respectively. For the datasets of yeast, *E.coli* and *C.elegans*, three-fifths of samples respectively from the positive dataset and negative dataset were randomly chosen as the training set. So the training sets of yeast, *E.coli* and *C.elegans* consisted of 7130, 8344, 4836 protein pairs respectively. Because human and *Drosophila* datasets contained a very large amount of samples, 20000 human protein pairs were randomly selected to construct the human training set and 15000 from *Drosophila* data set as the drosophila training set. The test sets of humans, yeast, *Drosophila*, *E.coli* and *C.elegans* were constructed with another 54054 human protein pairs, 4756 yeast protein pairs, 28950 *Drosophila* protein pairs, 5564 *E.coli* protein pairs and 3224 *C.elegans* protein pairs respectively. The protein pairs in test sets do not appear in the training sets.
