# Supplementary material for: PRED_PPI: a server for predicting protein-protein interactions based on sequence data with probability assignment
Source: BMC Res Notes. 2010 May 26;3:145. doi: 10.1186/1756-0500-3-145 (PMC2883990; doi:10.1186/1756-0500-3-145)
Supplement: Additional file 2 — Detailed prediction results. Additional File 2 is the supplemental data file providing detailed prediction results of the predictors built in this paper. It includes four tables. Table S1 lists the optimal values of C and γ for the predictors of five organism species. Table S2 shows the average prediction results for five organisms with probability threshold of 0.5, 0.6, 0.7, 0.8 and 0.9 respectively. Table S3 demonstrates the frequency distributions of the correctly predicted samples within different probability intervals and Table S4 shows the prediction performance of our method on the test set. [file 1756-0500-3-145-S2.DOC]

**Table S1.** The optimal values of *C* and γ for prediction models of five organism species.

|  | | |
| --- | --- | --- |
| Organism species | *C* | γ |
| *Human* | 32 | 0.03125 |
| *Yeast* | 32 | 0.03125 |
| *Drosophila* | 32 | 0.125 |
| *E.coli* | 32 | 0.03125 |
| *C.elegans* | 8 | 0.03125 |

*C* and γ are the two parameters of support vector machine. C is the regularization parameter and γ is the kernel width parameter.

**Table S2.** Average prediction results for five organisms with probability threshold of 0.5, 0.6, 0.7, 0.8 and 0.9 respectively.

| **A. For *Human* PPI prediction** | | | |
| --- | --- | --- | --- |
| Threshold | Sensitivity (%) | Specificity (%) | Accuracy (%) |
| 0.5 | 89.17 | 92.17 | 90.67 |
| 0.6 | 86.73 | 89.10 | 87.92 |
| 0.7 | 83.35 | 84.24 | 83.79 |
| 0.8 | 78.30 | 76.60 | 77.45 |
| 0.9 | 68.90 | 61.35 | 65.12 |
| **B. For *Yeast* PPI prediction** | | | |
| Threshold | Sensitivity (%) | Specificity (%) | Accuracy (%) |
| 0.5 | 88.17 | 89.81 | 88.99 |
| 0.6 | 85.07 | 86.02 | 85.55 |
| 0.7 | 80.32 | 80.73 | 80.53 |
| 0.8 | 73.91 | 72.84 | 73.38 |
| 0.9 | 62.86 | 58.86 | 60.86 |
| **C. For *Drosophila* PPI prediction** | | | |
| Threshold | Sensitivity (%) | Specificity (%) | Accuracy (%) |
| 0.5 | 99.53 | 80.65 | 90.09 |
| 0.6 | 99.33 | 77.25 | 88.29 |
| 0.7 | 99.06 | 83.00 | 86.03 |
| 0.8 | 98.79 | 67.19 | 82.99 |
| 0.9 | 98.17 | 57.16 | 77.66 |
| **D. For *E.coli* PPI prediction** | | | |
| Threshold | Sensitivity (%) | Specificity (%) | Accuracy (%) |
| 0.5 | 95.11 | 90.35 | 92.73 |
| 0.6 | 93.76 | 87.58 | 90.67 |
| 0.7 | 92.18 | 83.11 | 87.65 |
| 0.8 | 89.58 | 76.41 | 82.99 |
| 0.9 | 84.54 | 63.81 | 74.18 |
| **E. For *C.elegans* PPI prediction** | | | |
| Threshold | Sensitivity (%) | Specificity (%) | Accuracy (%) |
| 0.5 | 96.46 | 98.55 | 97.51 |
| 0.6 | 95.84 | 98.14 | 96.99 |
| 0.7 | 95.26 | 97.41 | 96.33 |
| 0.8 | 94.16 | 96.25 | 95.20 |
| 0.9 | 92.46 | 93.47 | 92.97 |

**Table S3.** The frequency distributions of the correctly predicted samples within different probability intervals.

| Probability interval | *Human* | *Yeast* | *Drosophila* | *E.coli* | *C.elegans* |
| --- | --- | --- | --- | --- | --- |
| 0.5<*P*<0.6 | 3.03% | 3.87% | 2.00% | 2.22% | 0.47% |
| 0.6≤*P*<0.7 | 4.55% | 5.65% | 2.51% | 3.26% | 0.73% |
| 0.7≤*P*<0.8 | 7.00% | 8.03% | 0.37% | 5.02% | 1.16% |
| 0.8≤*P*<0.9 | 13.59% | 14.06% | 8.91% | 9.51% | 2.30% |
| 0.9≤*P*≤1 | 71.83% | 68.39% | 86.21% | 79.99% | 95.34% |

*P*: probability.

**Table S4. The prediction performance of our method and the method of Shen *and others* (2007) on the original test set and independent test set respectively.**

| Predciton probability | Correctly predicted PPIs | | | |
| --- | --- | --- | --- | --- |
|  | Our method | | The method of Shen et al. | |
|  | Original set | Independent test | Original set | Independent test |
| ≧0.9 | 1885 | 1678 | 983 | 852 |
| ≧0.8 | 82 | 77 | 179 | 165 |
| ≧0.7 | 42 | 41 | 125 | 113 |
| ≧0.6 | 28 | 28 | 97 | 90 |
| >0.5 | 23 | 22 | 88 | 81 |
| Total | 2060 | 1846 | 1472 | 1301 |
| ≦0.5 | 141 | 137 | 729 | 682 |
| Total | 2201 | 1983 | 2201 | 1983 |
